# Bacterial cytological profiling reveals interactions between jumbo phage φKZ infection and cell wall active antibiotics in *Pseudomonas aeruginosa*

**DOI:** 10.1371/journal.pone.0280070

**Published:** 2023-07-07

**Authors:** Hannah Tsunemoto, Joseph Sugie, Eray Enustun, Kit Pogliano, Joe Pogliano

**Affiliations:** Division of Biological Sciences, University of California, San Diego, San Diego, CA, United States of America; Bhabha Atomic Research Centre, INDIA

## Abstract

The emergence of antibiotic resistance in bacteria has led to the investigation of alternative treatments, such as phage therapy. In this study, we examined the interactions between the nucleus-forming jumbo phage ФKZ and antibiotic treatment against *Pseudomonas aeruginosa*. Using the fluorescence microscopy technique of bacterial cytological profiling, we identified mechanism-of-action-specific interactions between antibiotics that target different biosynthetic pathways and ФKZ infection. We found that certain classes of antibiotics strongly inhibited phage replication, while others had no effect or only mildly affected progression through the lytic cycle. Antibiotics that caused an increase in host cell length, such as the cell wall active antibiotic ceftazidime, prevented proper centering of the ФKZ nucleus via the PhuZ spindle at midcell, leading us to hypothesize that the kinetic parameters of the PhuZ spindle evolved to match the average length of the host cell. To test this, we developed a computational model explaining how the dynamic properties of the PhuZ spindle contribute to phage nucleus centering and why some antibiotics affect nucleus positioning while others do not. These findings provide an understanding of the molecular mechanisms underlying the interactions between antibiotics and jumbo phage replication.

## Introduction

Jumbo phages create many complex protein structures to aid in replication but many mechanistic details remain unexplored. We have recently shown that several *Pseudomonas* jumbo phages 201φ201 [1], ФPA3 [2], and ФKZ [3, 4] have a complex life cycle involving both a cytoskeletal element composed of a phage-encoded tubulin (PhuZ) and a nucleus like structure that encapsulates phage DNA [5–10]. At the onset of infection, phage DNA is injected into the cell and immediately transcribed by a virion-packaged, multi-subunit phage-encoded RNA polymerase [4]. One of the first viral proteins to be abundantly expressed assembles a proteinaceous shell around the phage DNA to establish a compartment, the phage nucleus, within which phage DNA replicates. The phage nucleus contains the proteins necessary for DNA transcription, replication, recombination, and repair, while ribosomes and metabolic enzymes remain in the bacterial cytoplasm. This barrier is formed by the protein chimallin, which self-assembles into a square lattice containing 2nm sized pores that are big enough to allow the diffusion nucleotides yet small enough to maintain subcellular protein compartmentalization [11]. This remarkable subcellular organization provides a dedicated compartment rich in enzymes needed for DNA replication and affords protection from host defenses by excluding Cas and restriction enzymes [12, 13].

Once the phage nucleus is established, it is centered along the cell length by filaments of the PhuZ spindle. PhuZ is a tubulin-like protein that polymerizes to form triple-stranded filaments at each end of the cell [6, 8]. Early during the infection cycle, PhuZ filaments display dynamic instability, during which they undergo cycles of polymerization and depolymerization [8]. As filaments grow from one end of the cell, they make contact with the phage nucleus and push it towards the midcell. The GTPase activity of PhuZ is essential for dynamic instability and phage nucleus centering. Mutation of a conserved aspartic acid involved in coordinating an Mg^2+^ ion that is essential for GTP hydrolysis prevents filament depolymerization and phage nucleus centering [8, 14]. Late in the infection cycle, PhuZ filaments deliver newly formed capsids to the phage nucleus and simultaneously rotate the phage nucleus, which allows for an even distribution of capsids around the surface of the phage nucleus [5–7]. Thus, the phage nucleus and spindle are key structures that establish complex subcellular organization during viral replication and may play an important role in speciation of nucleus-forming viruses such as ФKZ [10].

The ability to find the cell midpoint is a critical feature among diverse biological systems. Many rod-shaped bacteria, for example, divide precisely in the middle of the cell, after the cell has reached twice its length. The Min system was first described in *Escherichia coli* by Adler et al. in 1967 [15]. The Min system facilitates proper positioning of the tubulin-like FtsZ ring at the cell center by preventing the formation of the divisome complex at the cell poles [16–22]. While we know the PhuZ spindle is responsible for pushing the *Pseudomonas* phage nucleus towards midcell during phage infection [8, 14], we currently lack insight into the important parameters and constraints that might dictate the behavior of the system.

Here we investigated the interplay between antibiotics and nucleus forming jumbo phage replication, focusing on antibiotics that are commonly used in treatment against *P*. *aeruginosa*. *P*. *aeruginosa* has the largest genome of the ESKAPEE group of pathogens and according to the Centers for Disease Control and Prevention, it is the causative agent of approximately 33,000 healthcare-associated infections annually in the United States alone [23]. In addition to its intrinsic antibiotic resistance mechanisms, *P*. *aeruginosa* can form biofilms, especially in the lungs of people with cystic fibrosis or on surgical implants, which can hinder successful antibiotic treatment [24–26]. These difficulties make the application of phage therapy, in tandem with antimicrobial treatments, for *P*. *aeruginosa* infections particularly appealing [27]. There has been an increase in successful uses of phage therapy against multi-drug resistant infections reported in the last few years [26, 28, 29].

Phage therapy is currently considered a treatment of last resort, so patients are most likely already receiving one or more antibiotics despite their lack of efficacy in clearing the infection. This highlights the necessity for understanding how antibiotics and phages interact. Concurrent treatments with antibiotics and phage can simultaneously reveal antagonistic combinations that are ultimately detrimental to the patient as well as provide insights into details of phage replication processes. Previous research has demonstrated phage-antibiotic synergy (PAS) in a variety of species, including *P*. *aeruginosa* [24, 30–34], but the underlying mechanisms are unclear.

Here, we explored how antibiotics commonly used to treat *Pseudomonas* infections affect phage replication during a single round of infection by the jumbo phage ФKZ. Given the remarkable subcellular organization of nucleus-forming jumbo phage, it was unclear how antibiotics typically used during phage therapy would affect their life cycle. We specifically addressed how cell wall antibiotics affect phage infection and formation of the phage nucleus and spindle. We used a fluorescence microscopy tool termed bacterial cytological profiling (BCP) to study PAS. BCP can classify the mechanism of action (MOA) of an antibiotic based on cytological changes of the bacterial cell, such as cell wall shape or DNA morphology, during treatment in both Gram-negative and Gram-positive species [35–39]. The benefit of BCP is the ability to observe both phenotypic changes in phage infection and cytological shifts due to antibiotics at the single cell level.

We show that antibiotics targeting different biosynthetic pathways have different effects on the progression of ФKZ infection. We specifically focused on determining which antibiotics, if any, might affect positioning of the phage nucleus at midcell. We found cell-elongating antibiotics had a dramatic effect on the ability of the phage nucleus to be centered. We developed a simple computational model to show how dynamically unstable filaments might be capable of positioning the phage nucleus at midcell and show that cell length is a key parameter that determines the efficiency of centering by the PhuZ spindle. We experimentally confirmed our model by showing phage nucleus mispositioning matches the model’s predictions when cell length is altered. Our results suggest that the PhuZ spindle has evolved kinetic parameters that allow it to optimally position the phage nucleus in cells of a given length and provide insight into how clinically used cell wall antibiotics affect nucleus-forming jumbo phage replication.

## Results

### Antibiotic specific effects on the percentage of cells infected by φKZ

**Fig 1:**
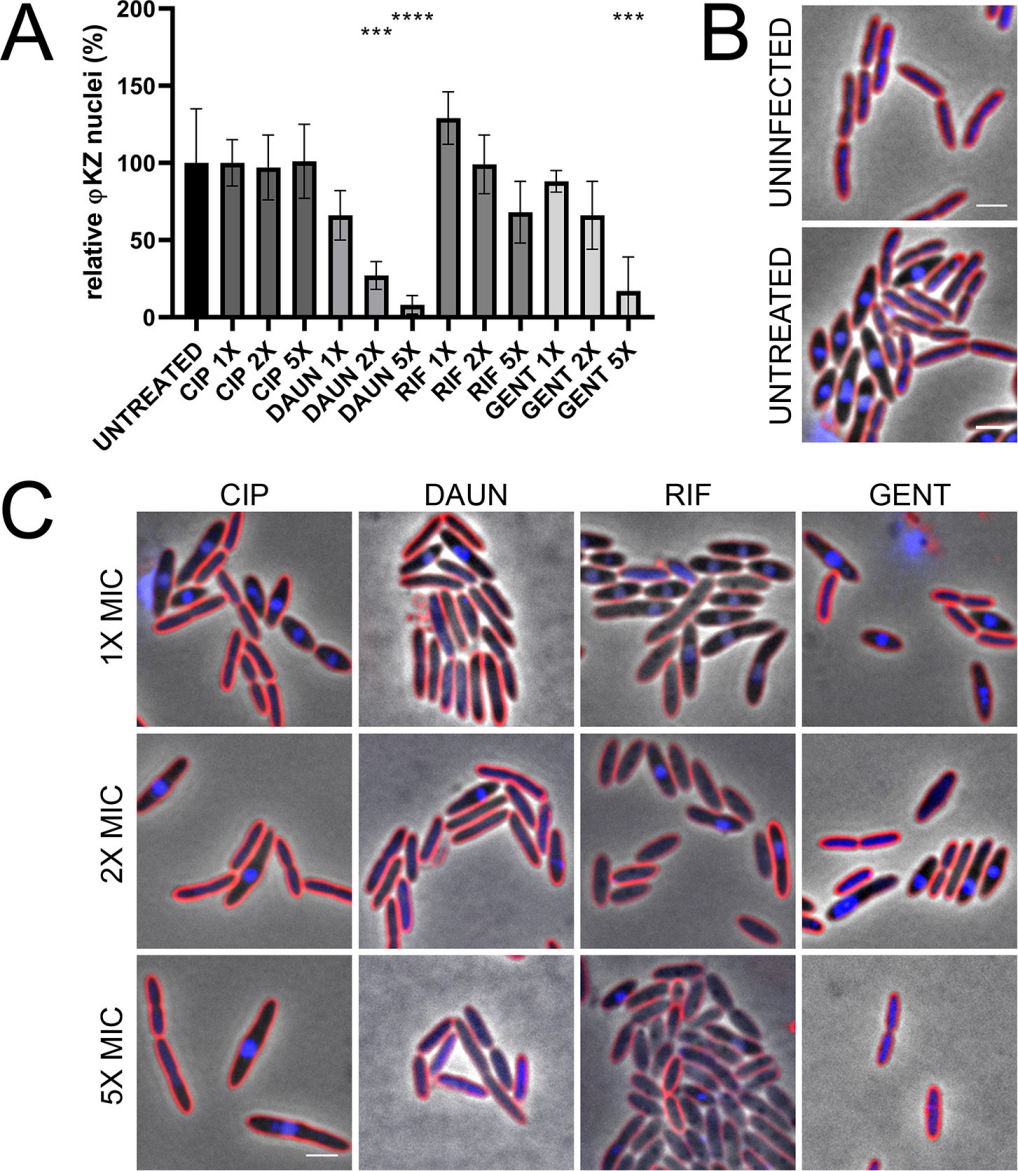
Treatment of *P*. *aeruginosa* K2733 with different antibiotics show differential φKZ infection related to MOA and host cell phenotype at 30 min post-infection. (A) Quantification of phage infection (presence of distinct phage nuclei) under treatment conditions, relative to the untreated infected control. Error bars represent standard deviation of biological triplicates. *** = p < 0.0005, **** = p < 0.00005 (B) Microscopy of uninfected and untreated infected controls, and (C) treated infected samples: ciprofloxacin (CIP), daunorubicin (DAUN), rifampicin (RIF), and gentamicin (GENT). Cell membrane strained with FM4-64 (red) and DNA stained with DAPI (blue). Scale bar represents 2 μm.

**Fig 2:**
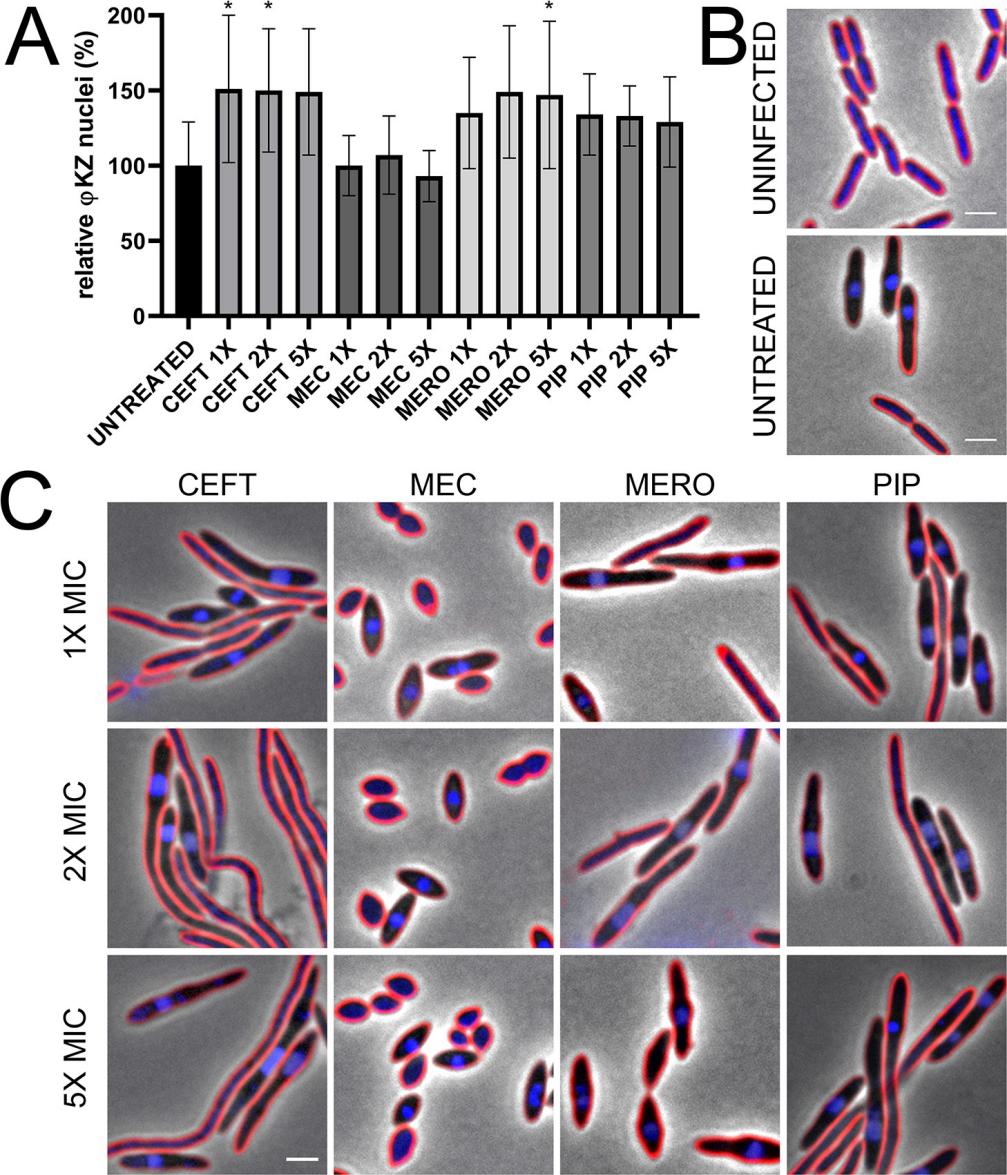
Treatment of *P*. *aeruginosa* K2733 with cell wall active antibiotics show differential φKZ infection related to MOA and host cell phenotype at 30 min post-infection. (A) Quantification of phage infection (presence of distinct phage nuclei) under treatment conditions, relative to the untreated infected control. Error bars represent standard deviation of biological triplicates. * = p < 0.05 (B) Microscopy of uninfected and untreated infected controls, and (C) treated infected samples: ceftazidime (CEFT), mecillinam (MEC), meropenem (MERO), and piperacillin (PIP). Cell membrane strained with FM4-64 (red) and DNA stained with DAPI (blue). Scale bar represents 2 μm.

We surveyed commonly used antibiotics against *P*. *aeruginosa* infections that had various mechanisms of action (MOAs) to determine whether specific classes of inhibitors hinder phage replication, potentially diminishing the effectiveness of adding phage to the treatment, or if any classes promote infection and act synergistically as previously reported [34]. We studied jumbo phage ФKZ replication in *P*. *aeruginosa* K2733 during a single round of infection to identify antibiotics that might promote or inhibit completion of the phage life cycle. The *P*. aeruginosa K2733 strain lacks components of the major Resistance-Nodulation-Division (RND) pumps involved in the efflux of antibiotics of the bacterial cell. We chose to use this sensitized strain to increase the diversity of biosynthetic pathways targetable by antibiotics. We treated cells at concentrations ranging from 1X to 5X the relative minimum inhibitory concentration (MIC) for each antibiotic for 30 min at 37°C prior to infection with the jumbo phage ФKZ. We used BCP to visualize the effect of the antibiotic treatment on the morphology of the bacterial cell and simultaneously visualize the establishment of a phage nucleus at 30 minutes post infection (mpi), a total of 60 minutes of antibiotic treatment. Infection of the bacterial cell can be visualized via bright DAPI staining of the phage nucleus (Figs 1B, 1C, 2B and 2C). Images were quantitated and the percentage of the population containing a phage nucleus, an indicator of phage replication, was expressed relative to the untreated control (Figs 1A and 2A). As expected, we found gentamicin (GENT) which targets protein synthesis and prevents phage protein expression, and daunorubicin (DAUN), which non-specifically intercalates into DNA, strongly inhibited the appearance of a phage nucleus at 30 mpi in a concentration dependent manner (Fig 1A). In contrast, rifampicin (RIF) which targets host RNA polymerase but not the phage RNA polymerase, had little effect on the progression of phage infection, as expected since ФKZ encodes two multi-subunit RNA polymerases that are resistant to RIF [4, 40, 41]. Pretreatment with ciprofloxacin (CIP), which targets DNA gyrase, also had no effect on phage infection, which is in agreement with previous work [33].

It has been previously reported that the β-lactam antibiotics ceftazidime (CEFT) and piperacillin (PIP) have synergistic effects with KPP22 phage infection against *P*. *aeruginosa* [34]. We therefore examined the potential for synergy with ФKZ during a single round of infection by quantitating the percentage of cells that became infected in the presence of cell wall synthesis inhibitors that cause specific changes in host cell morphology relative to the untreated controls (Figs 2 and S1). We chose four β-lactams that have affinities for different penicillin-binding proteins (PBP): CEFT and PIP inhibit PBP3 which catalyzes peptidoglycan cross-linking during cell division, causing elongated cells [42]; meropenem (MERO) inhibits PBP2 and PBP4, resulting in elongated cells that bulge at midcell; and mecillinam (MEC) specifically binds to PBP2 [43] and causes oval-shaped cells (Figs 2C and S1). None of the four cell wall active antibiotics produced a strong effect on infection rates, with only two concentrations of CEFT and one concentration of MERO causing a small but statistically significant increase (30 to 50%) of infections (Fig 2A).

### Treatment with antibiotics that cause cell elongation leads to mispositioning of the φKZ phage nucleus

**Fig 3:**
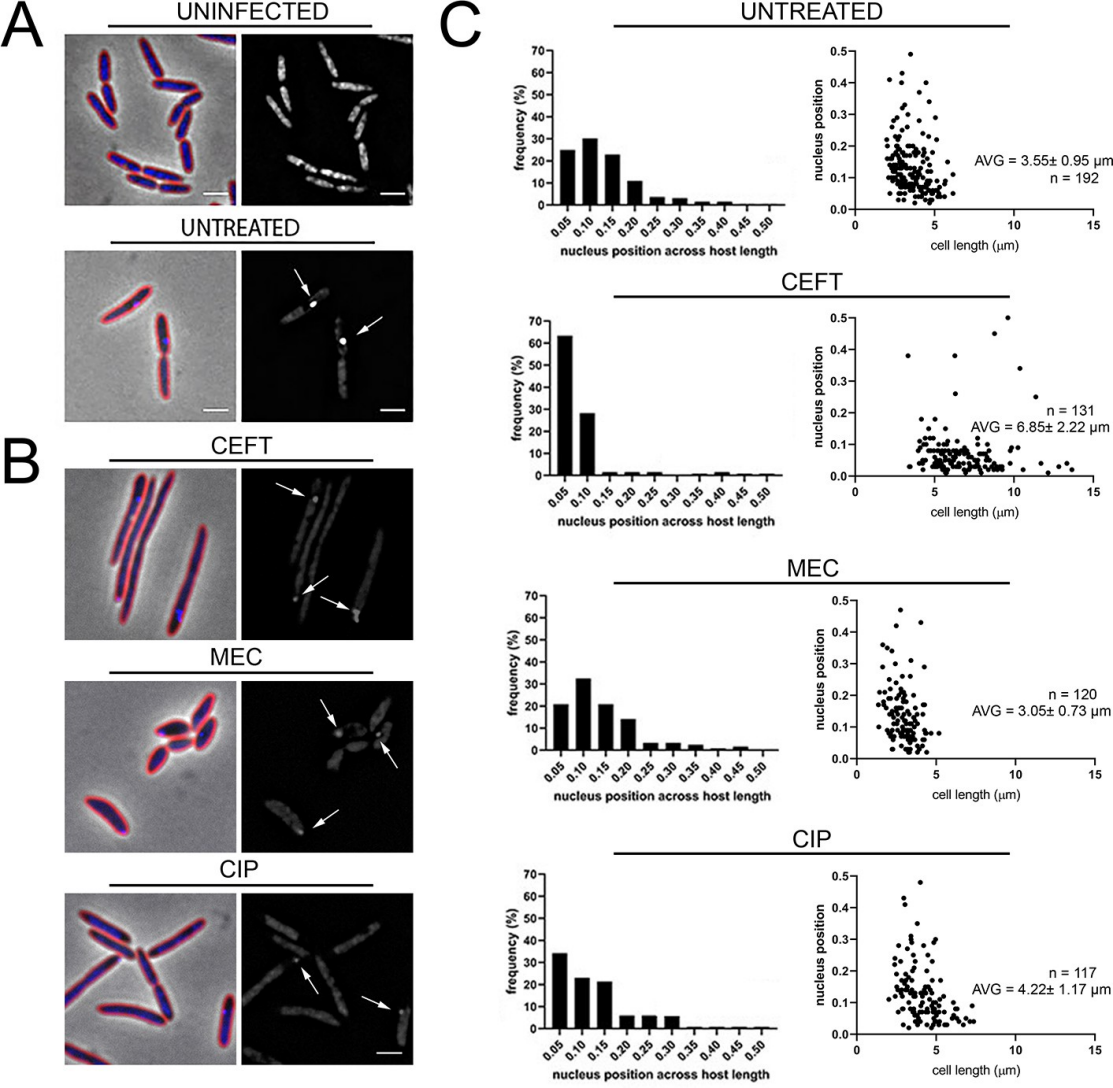
Treatment with CEFT increases early φKZ binding and infection. (A) Microscopy of uninfected and untreated infected controls, and (B) treated infected samples with 5X MIC at 5 min post-infection. Cell membrane strained with FM4-64 (red) and DNA stained with DAPI (blue). Scale bar represents 2 μm. White arrows indicate phage DNA at early stages of infection. (C) Nucleus position across *P*. *aeruginosa* host length of untreated and treated (5X MIC) infected samples. Histograms show early phage nucleus position across normalized host cell length. Scatterplots show early phage nucleus position across relative host cell length. Average cell length for each treatment condition shown.

During our study of cell wall elongating antibiotics, we noticed a potential connection between host cell length and the position of the phage nucleus. A key function of the PhuZ spindle encoded by ФKZ and related *Pseudomonas* jumbo phages is to position the phage nucleus at the center of the cell [8, 9, 14]. At the onset of infection (5 mpi), phage DNA can be observed at the cell poles in >85% (n = 192) of untreated infections using DAPI staining (Fig 3A and 3C). During treatment with either CIP, CEFT, or MEC, the injected phage DNA can still be observed at the poles of the host cell (Fig 3B and 3C). These results taken together with prior studies show that phage DNA is injected near the cell poles and suggest that the receptor for ФKZ is likely located near the cell poles.

**Fig 4:**
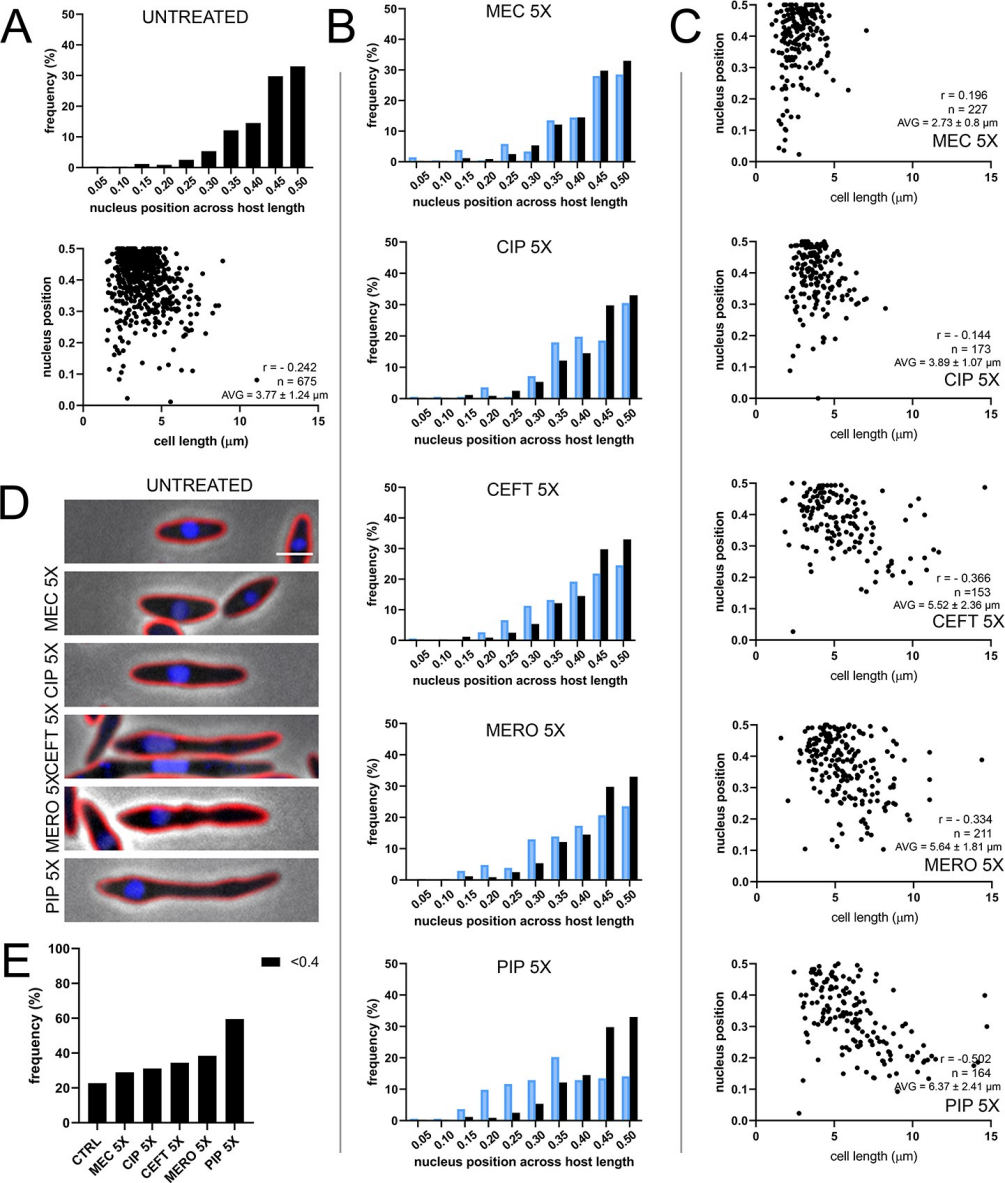
Histogram of nucleus position across normalized *P*. *aeruginosa* host cell length for (A) untreated infected control and for (B) treated samples at 5X MIC, in blue, at 30 min post-infection. Black bars represent untreated infected control and are included for comparison. Scatterplots show nucleus position for across relative *P*. *aeruginosa* host cell length for (A) untreated infected control and for (C) treated samples at 5X MIC, along with average cell length for each treatment condition. Correlation coefficients between average cell length and nucleus position (r) shown for each treatment condition. (D) Representative cell images of untreated infected controls and treated infected samples. Cell membrane strained with FM4-64 (red) and DNA stained with DAPI (blue). Scale bar represents 2 μm. (E) Frequency of nucleus position out of the middle 20% of the host cell for untreated and treated samples at 5X MIC.

The PhuZ spindle normally repositions the replicating phage DNA contained inside the phage nucleus towards midcell using dynamically unstable filaments [8, 9, 14]. As expected, by 30 mpi, phage DNA is primarily localized near the midpoint of the host cell (Figs 1B, 2B and 4A, [6–8, 14]). Fig 4B shows histograms of phage nucleus position normalized to cell length and compared to the DMSO control (black) for each antibiotic at 5X MIC (blue). During treatment with cell wall synthesis inhibitors such as CEFT or PIP, as the length of the cell is increased, the position of the phage nucleus at 30 mpi becomes increasingly off-centered compared to untreated controls (Figs 2C, 4B, 4D and 4E). MEC and CIP are relatively unaffected, while PIP treatment caused the largest average shift from center as well as the greatest increase in cell length. As the average cell length increased, the percentage of nuclei that were positioned away from the center increased (Fig 4B–4E). For example, within DMSO treated cells, approximately 24% of phage nuclei are off-center (outside of the 20% middle of the cell), whereas in piperacillin treated cells, 60% of phage nuclei are off-center (Fig 4E). These results suggest that either cell length itself plays a factor in spindle positioning of the phage nucleus or the antibiotics have perturbed spindle assembly.

**Fig 5:**
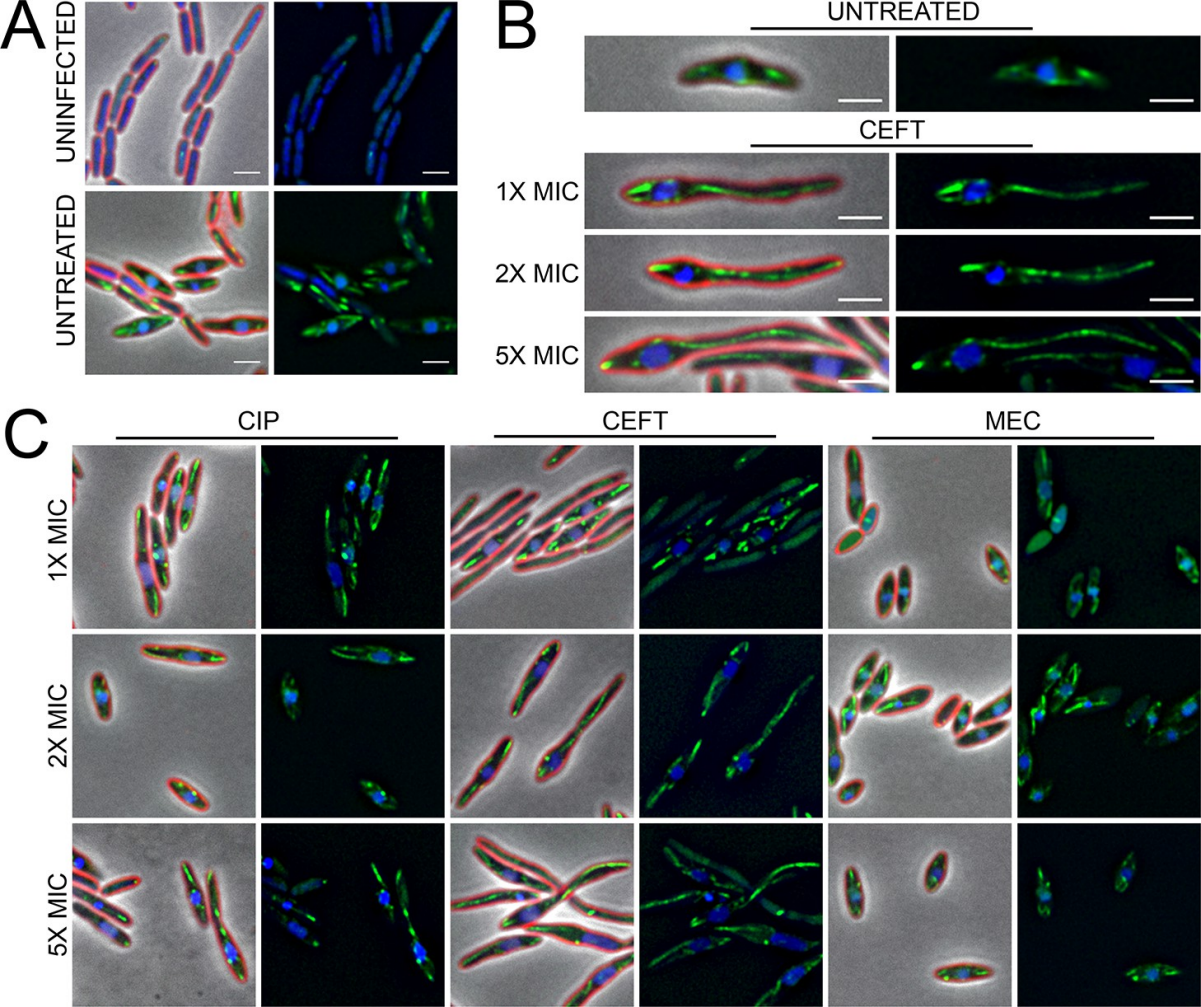
Antibiotic treatment leads to aberrant PhuZ filament and spindle dynamics at 30 min post-infection. (A) Microscopy of uninfected and untreated infected controls. (B) Representative cell images of untreated infected controls, and CEFT treated infected samples at all concentrations. (C) Microscopy of treated infected samples. Cell membrane strained with FM4-64 (red) and DNA stained with DAPI (blue). GFP-PhuZ (green) under 0.1% arabinose induction. Scale bar represents 2 μm.

One possible explanation for mispositioning could be the inability to assemble the PhuZ spindle. To determine if treatment with these antibiotics prevented spindle assembly, we treated *P*. *aeruginosa* expressing a low level of a GFP-PhuZ fusion protein [6] with increasing concentrations of CEFT, MEC, or CIP prior to infection with ФKZ (Fig 5). In the absence of phage infection, GFP-PhuZ occasionally formed small foci but did not assemble filaments due to its low level of expression (Fig 5A) [6]. In the untreated infection condition, the PhuZ spindle formed at both poles of the cells reaching towards the phage nucleus and positioned it at approximately the cell midpoint (Fig 5A). During treatment with CEFT and CIP, filaments still formed on each side of the nucleus in the elongated cells, except now one PhuZ filament frequently extended further than the other (Fig 5B and 5C). In comparison, for all concentrations of MEC, PhuZ filaments were observed, and the phage nuclei were positioned at midcell (Fig 5C). With both the properly positioned and mispositioned nuclei, PhuZ filaments appeared to be assembled. Therefore, nucleus mispositioning was not due to an inhibition of spindle formation.

**Fig 6:**
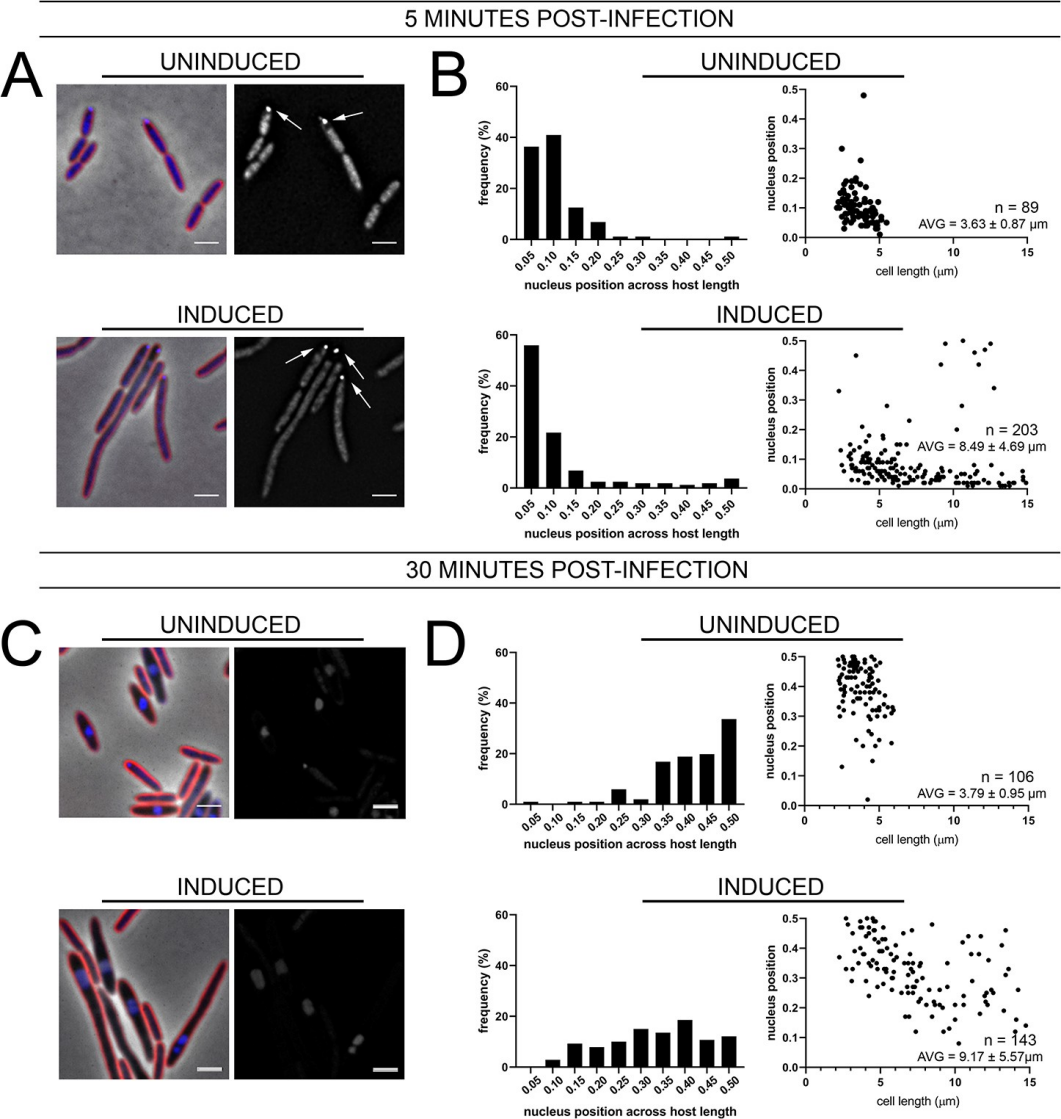
Over-expression of the cell division inhibitor protein sulA leads to increased cell length but not increased φKZ infection rates. (A,C) Microscopy of infected uninduced or induced sulA cells at (A) 5 min or (C) 30 min post-infection. Cell membrane strained with FM4-64 (red) and DNA stained with DAPI (blue). Scale bar represents 2 μm. White arrows indicate phage DNA at early stages of infection. (B,D) Histogram of phage nucleus position across normalized host cell length at (B) 5 min or (D) 30 min post-infection. Scatterplots (D) show nucleus position for across relative *P*. *aeruginosa* host cell length at 30 min post-infection for uninduced or induced sulA cells, along with average cell length for induced and uninduced conditions.

Our results showing that antibiotics targeting different pathways resulted in cell elongation and increased phage nucleus mispositioning suggested that cell length might be a key factor in phage nucleus positioning. If this were true, we predicted that other methods of inhibiting cell division should also affect phage nucleus positioning. To test this model, we studied phage nucleus positioning after expressing SulA from a plasmid in *P*. *aeruginosa* K2733 (Fig 6). SulA prevents cell division by binding to the bacterial tubulin homolog FtsZ and inhibiting the assembly of the Z ring [44]. We quantitated the extent to which SulA expression affected cell length, the rates of ФKZ infection, and phage nucleus positioning. At 30 mpi, when SulA was not induced, cells exhibited a range of cell length distributions similar to wild-type cells (Fig 6A and 6C, top), but cells became very elongated when SulA was induced (Fig 6A and 6C, bottom). When uninduced or induced SulA populations were infected and examined at 5 mpi, the nascent phage nucleus was positioned near the cell pole as in wild-type cells (Fig 6A and 6B). This demonstrated that SulA expression did not affect the initial site of DNA injection. In contrast, when examined after 30 mpi (Fig 6C and 6D), there was a dramatic effect on phage nucleus positioning (Fig 6D, bottom). Rather than being positioned near midcell as in the uninduced samples (Fig 6D, top), the phage nuclei were uniformly distributed throughout the cells (Fig 6D, bottom) with the exception that they were excluded from the cell poles. Taken together, our results revealed for the first time a possible connection between host cell length and the positioning of the phage nucleus at midcell.

### Stochastic modeling of PhuZ filaments

**Fig 7:**
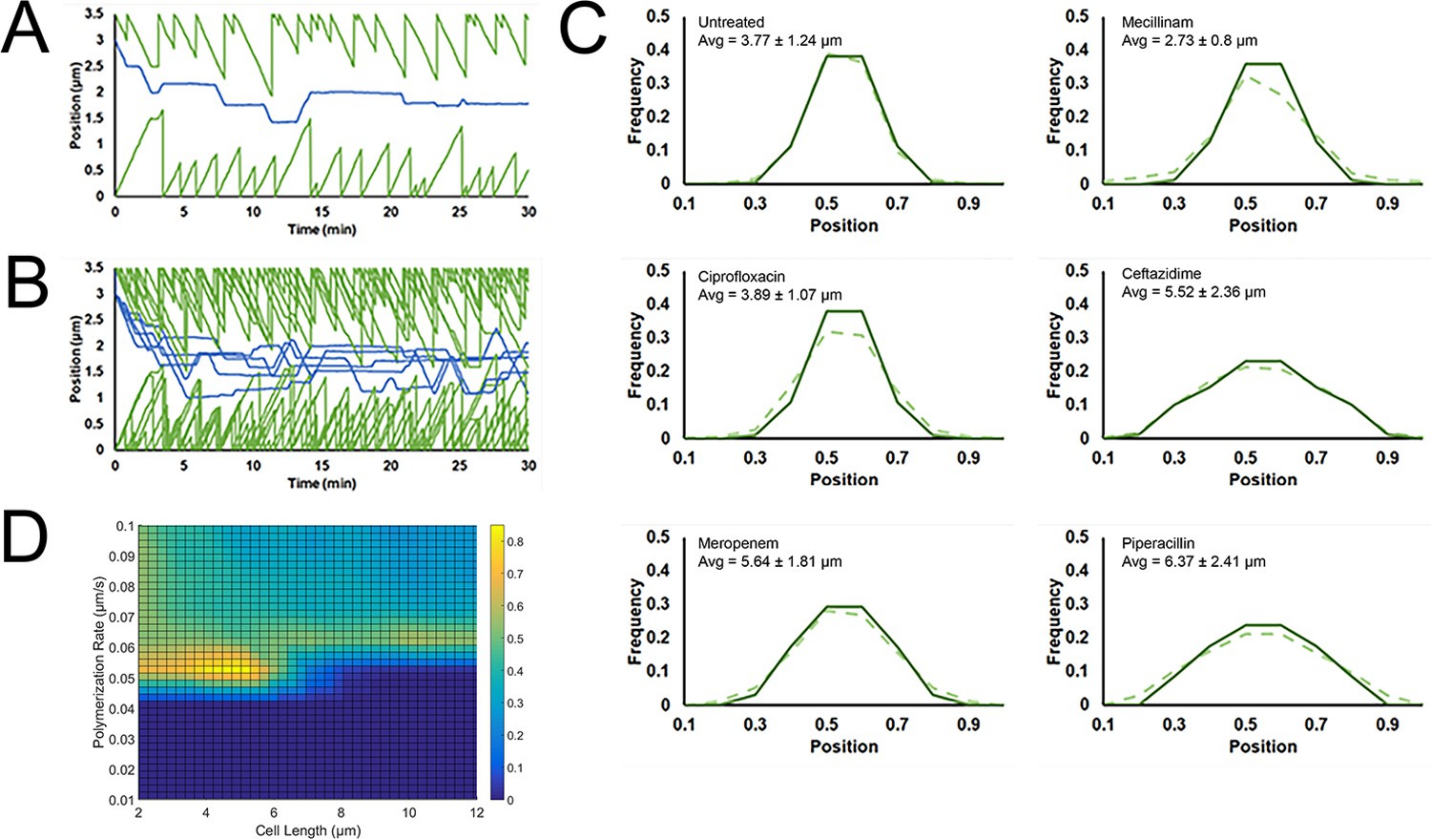
Computational modeling of PhuZ dynamics under antibiotic treatment. (A) A single run of the model showing filament end positions in green and the position of the phage nucleus center in blue. The cell length was set at 3.5 μm and the simulation ran for 30 minutes. (B) Five overlapping traces of the model using parameters identical to (A) provide a visualization of the stochastic behavior of the model. (C) Histograms of phage nucleus position after 30 minutes has elapsed for both the model (solid line) and the measured data (dotted line). All model parameters were kept the same with the exception of cell length which was sampled from a normal distribution with mean and standard deviation given by each treatment condition. (D) Heatmap indicating combinations of cell length and polymerization rate that lead to centering of the phage nucleus. Yellow indicates that a high (>80%) percentage of the phage nuclei positions were located within the central 20% of the cell while blue indicates a low percentage. The effect of all parameters reveals a narrow window in which the phage nucleus can be effectively centered.

To understand the connection between cell length and phage nucleus positioning, we developed a computational model to describe how PhuZ filaments can center the nucleus in a wild-type cell and how perturbing cell length might influence centering. Previous work in our lab demonstrated that DNA objects can be centered in anisotropic cells when the rates of filament polymerization and depolymerization matched a specific range of cell lengths [8, 45]. Therefore, a 1D stochastic model of PhuZ filaments was created to determine if cell length changes could explain the various changes to phage nucleus positioning observed across different conditions. Visualizations of filament and nucleus positions as modeled over 30 minutes are shown in Fig 7A and 7B.

Sampling cell length from a Gaussian distribution given by the mean and standard deviations of various treatment conditions shows a good fit to measured phage nuclei position distributions indicating that cell length could be a determining factor in phage nucleus mispositioning (Fig 7C). As shown in Fig 7C, the model (solid line) can reproduce the measured distributions of phage nucleus positioning (dashed line) indicating that cell length alone is sufficient to explain the observed behavior. To illustrate this further, we explored the phase space by varying cell length and polymerization rate and observed the effect this had on nucleus positioning (Fig 7D) and found nucleus positioning at midcell exists for a narrow window of cell lengths and polymerization rates. These results suggest that the phage co-evolved with the host cell to optimize the rates of PhuZ polymerization, depolymerization and cell length in such a way as to properly center the phage nucleus. During treatments that lead to aberrant cell length this balance is disrupted as the spindle properties do not adapt, leading to mispositioning of the phage nucleus.

## Discussion

These studies demonstrated the complexity of antibiotic-phage interactions at the single-cell level. Using BCP, we visualized interactions between phages and antibiotics during infection which allowed us to generate potential mechanistic explanations for the observed interactions. PAS has been demonstrated in a variety of bacterial species [24, 31–34], but the underlying mechanisms for these interactions remain uncharacterized and synergistic interactions have not been observed for each combination of phage and antibiotic. ß-lactam antibiotics have previously been shown to synergize with the *Pseudomonas* phages KPP21, KPP23, and KPP25 [34], and in contrast, no synergy was observed with the fluoroquinolone ciprofloxacin [33]. In this study, we observed that the DNA intercalator DAUN and the protein synthesis inhibitor GENT prevented ФKZ phage nucleus formation in a dose-dependent manner. These antibiotics strongly block phage replication and potentially reduce phage amplification during treatment, thereby counteracting any potential benefits from combination therapy. In contrast, using our single cell studies of ФKZ and *P*. *aeruginosa* to search for evidence of enhancement of the phage infection with ß-lactam antibiotics, we saw little to no evidence that any of the ß-lactams tested increased infection rates.

While we did not detect a dramatic effect of cell wall active antibiotics on phage infection rates, we observed consistent injection of phage DNA at the poles of the bacterial cells, regardless of treatment (Fig 3), suggesting the location of the ФKZ receptor. After forming at the cell pole, the phage nucleus migrates to midcell (Fig 4A). In cases where the length of the host cell was increased due to antibiotic treatment, there were greater instances of mispositioning of the phage nucleus away from midcell (Fig 4B, 4C and 4E).Mispositioning was more pronounced when we artificially triggered cell elongation by inducing the cell division inhibitor SulA (Fig 6D). These studies uncovered a potential connection between cell length and the ability of the PhuZ spindle to position the phage nucleus at midcell.

We previously described a phage encoded tubulin-based system that can position a protein shell containing replicating phage DNA at the center of the host cell [8, 9, 14]. Our observation of mispositioning in the presence of cell elongating antibiotics might have suggested the lack of spindle formation or the formation of short spindles, but these cells contained long, asymmetric spindles, some reaching more than 7 microns in length (Fig 5). While it would not be surprising for phage nuclei to be mispositioned in very long cells (more than 10μm in length), we found that cells that were only slightly longer than normal (5 to 10 μm in length) were also mispositioned (Figs 4 and 6). We therefore hypothesized that the kinetic parameters of the PhuZ filaments might be tuned to position the phage nucleus in cells of only a certain length. To explore this possibility, we developed a computational model of phage nucleus position that incorporated known kinetic parameters and compared the results of the model to the experimental data (Fig 7). We determined that the elongation of *P*. *aeruginosa* cells is likely the main factor that contributes to the observed mislocalization of the phage nucleus away from midcell (Figs 4–7). Our computational model correctly predicted DNA centering under normal conditions and when cell length is strongly affected by the antibiotics. Centering is only achieved computationally when estimated parameters of polymerization and depolymerization rates fall within a certain range for a cell of a given length, providing insight into the molecular basis for PhuZ spindle centering of the phage nucleus in bacteria. These findings support our prior work on dynamically unstable bacterial actins, where the interplay between rates of polymerization, depolymerization, and cell length determined whether a DNA object was positioned at the cell center or positioned at the cell poles [8, 45, 46].

Spatial information is important for the positioning of molecular complexes within a cell, such as at the pole or midpoint, and is required in all biological systems from eukaryotes to microbial organisms [16, 20–22, 47–49]. Dynamic instability of cellular filaments has been shown to be the driving force required for centering of DNA objects in bacteria and for phages [8, 9, 14, 45, 46]. The work presented here also highlights host cell length as an intrinsic determinant of PhuZ spindle positioning of the phage nucleus at midcell. Our data suggests that the phage replication machinery co-evolved with and is optimized to the average length of the *Pseudomonas* host cell. We observed that antibiotic-induced increase in cell length no greater than 2-fold led to drastically inability of the PhuZ filaments to properly center the phage nucleus within the host cell. The computational model developed in this study was able to mimic experimentally confirmed nucleus positions based on three general parameters, host cell length and polymerization/depolymerization rates of the PhuZ filament, thereby expanding our understanding of the factors influencing nucleus-forming phage infection. Perturbations of host cell length by antibiotics drastically impeded the ability of PhuZ filaments to properly center the phage nucleus.

The *E*. *coli* phage Goslar was recently shown to form a chimallin based phage nucleus that compartmentalizes phage replication similar to the *Pseudomonas* phages [50]. While Goslar also encodes a PhuZ tubulin that forms a set of vortex-like filaments that rotate the phage, unlike *Pseudomonas* phages, Goslar’s phage nucleus is not positioned at midcell. Injection of Goslar phage DNA occurs at any point along the bacterial cell surface, so it does not require a system dedicated for moving the nucleus away from the cell pole but rather only for rotation [50]. In comparison, we observed ФKZ DNA injection exclusively at the poles of the *P*. *aeruginosa* cell (Fig 3), and the ФKZ phage nucleus requires the polymerization of the bipolar PhuZ filaments to push it into position from the poles to midcell (Fig 5). These differences demonstrate the co-evolution of phages and their tubulins to optimize replication within their respective hosts. We hypothesize that differences in receptors and positioning of the initial injection event for these two phages may be partly responsible for the different filament dynamics.

Overall, the data presented here also brings up additional questions for further study, including what role does the centering of the phage nucleus play in the efficiency of phage replication and does mispositioning of the phage nucleus impact phage particle production? While PhuZ is not essential for phage replication based on dominant negative [6, 7, 14] and gene deletion studies [51], PhuZ spindle rotates the phage nucleus and brings empty phage capsid heads to it for viral DNA packaging [5, 10]. It is possible that the asymmetric phage spindle observed during treatment with cell wall targeting antibiotics may lead to inefficient packaging of phage particles due to altered phage capsid transportation or changes in the rotation of the phage nucleus by the spindle. This study looked at the impact of antibiotic treatment on a single round of infection. Future experiments could measure the speed of rotation of the phage nucleus or the number of phage particles over multiple rounds of infections concurrent with antibiotic treatment to see if there is a negative effect on phage replication beyond the phenotypic changes in host and viral replication machinery observed in this study. Finally, fully understanding how jumbo phage replication is affected by antibiotics will ultimately provide guidance for the selection and design of compatible phage-antibiotic mechanistic pairs useful for phage therapy.

## Material and methods

### Bacterial strains, growth, bacteriophage preparation, and antibiotics

*Pseudomonas aeruginosa* strain K2733 (PAO1ΔmexB, ΔmexX, ΔmexCD-oprJ, ΔmexEF-oprN) was used in this study. The bacteria were grown in LB, with gentamicin for selection when necessary, at 37°C for all experiments. Preparations of antibiotics were performed according to the manufacturer’s recommendations. Jumbo phage φKZ lysate was prepared by adding 5 mL phage lysate and 100 mL fresh LB to 50 mL saturated ON culture of *P*. *aeruginosa* PAO1 and incubating at 37°C for at least 5 hours, or until visible clearance of culture. Cultures were centrifuged at 4000 rpm for 15 min and the supernatant filtered to remove bacterial cell debris. Titers were performed and the phage lysate was stored at 4°C until use.

*P*. *aeruginosa* strain K2733 expressing GFP-PhuZ was generated as detailed in Chaikeeratisak, et al [6]. GFP-PhuZ production was induced using 0.1% arabinose. SulA (Accession No: CP053028, from 2146814 to 2147299) was PCR amplified from K2733 strain with the primers that include 25 nucleotide overhang that is homologous to pHERD30T plasmid backbone (Forward Primer: ATTCTTTAAGAAGGAGAAATTCACCATGCAGACCTCCCACTCG, Reverse Primer: ACTCTAGAGGATCCCCGGTACCTCAACCCAGACGAATATTCAGGCTCTG). The PCR product was Agarose gel extracted and inserted into a linearized pHERD30T vector with NEBuilder HIFI DNA Assembly (NEB, E2621L). Electrocompetent K2733 strain was transformed with the plasmid and induced with 0.5% arabinose.

### Minimal inhibitory concentration (MICs) assays

MICs for all antibiotics (Table 1) were determined using the broth microdilution method [35]. In brief, overnight cultures of *P*. *aeruginosa* K2733 were diluted 1:100 in fresh LB and allowed to grow at 37°C with rolling until they reached an optical density at 600 (OD_600_) of ∼0.2, or early exponential phase. The bacterial culture was diluted to OD_600_ ∼ 0.05, and then diluted 1:100 into the appropriate wells of a 96-well plate containing serially diluted antibiotics. MICs were determined by OD_600_ readings after incubation at 37°C with shaking for 18–24 hours.

**Table 1:** Minimum inhibitory concentration (MIC) of antibiotics used in this study against *P*. *aeruginosa* K2733. Concentrations shown in μg/mL and represent biological duplicates.

DNA/RNA SYN		CELL WALL SYN	
**CIP**	0.013	**CEFT**	2
		**MEC**	6.4
		**MERO**	0.1
		**PIP**	0.5

### Fluorescence microscopy

Overnight cultures of *P*. *aeruginosa* K2733 were diluted 1:100 into fresh LB, with appropriate arabinose concentrations when required, and allowed to grow at 37°C with rolling until they reached an OD_600_ ∼0.12–0.15. 400 uL culture was then added to cultures tubes containing 1X, 2X, or 5X MIC of each antibiotic and incubated at 37°C with rolling for one hour, for antibiotic only controls, or 30 minutes, for subsequent phage infection. Lysate of the jumbo phage was then added to cultures, at a multiplicity of infection (MOI) of 5, before incubation at 37°C with rolling for an additional 30 minutes. Uninfected and untreated controls were done each experimental day. After infection, 4 uL culture was added to 4 uL dye mix containing 100 ug/mL FM4-64 and spotted onto pad slides containing 1.2% agarose in 20% LB with 0.2 ug/mL DAPI for microscopy. Microscopy was performed as previously described [35], with slight modifications. Excitation and emission settings were kept consistent for all replicate experiments.

#### Quantitation and statistical analysis of phage infection.

φKZ infection, defined as a distinct phage nucleus or the presence of a distinct phage DNA puncta upon injection within the host cell, was quantified manually using FIJI (ImageJ 1.51w). All microscopy experiments were performed in biological triplicate. Statistical analysis was conducted in GraphPad Prism 8.4.3. One-way ANOVA followed by Dunnett’s multiple comparisons test was used to determine significance differences between the untreated infected control and the treatment conditions. Statistical significance was defined as a p value of < 0.05. In quantitation graphs, p values indicated as follows: * ≤ 0.050,– 0.0100, ** ≤ 0.0100–0.0010.

### Stochastic modeling of PhuZ filaments

A 1D model of PhuZ filament movement was created by considering the net movement of a filament’s position to be a balance between polymerization, depolymerization and catastrophe. A simple equation to describe this movement in the positive direction is given as:

$$\frac{dx}{dt}=v_{polymerization}-v_{depolymerization}-v_{catastrophe}$$

The speed of polymerization, depolymerization and catastrophic depolymerization can both be sampled from a Gaussian distribution determined by the mean and standard deviation of parameters based on previous measurements and publications [25] as well as parameter optimizations to measured data.

The model consists of two filaments with initial positions at x = 0, L_c_, where L_c_ is the cell length and was run with a timestep of 0.01 seconds. All parameters related to movement of filaments get sampled from a Gaussian distribution and positions are updated if the random time sampling associated with their movement has passed. Catastrophe occurs after a given time, which is again sampled from a Gaussian distribution, after which time the speed of catastrophic depolymerization becomes non-zero. Simultaneous to the occurrence of catastrophe, a recovery time is generated which determines when catastrophic depolymerization again becomes zero. The position of the nucleus is initially set at 90% of the cell length and moves either by random diffusion for a sphere with a Stokes radius of 1 μm or by collisions with growing filaments. If a filament would move beyond the boundary of the phage nucleus in a given timestep, the nucleus position is pushed by an equal amount to prevent clipping of the filament into the nucleus. However, if the filament from the opposite pole is pushed to the boundary of the phage nucleus when this would occur, no movement from either nucleus or filaments takes place. In this condition, movement can only occur after catastrophe induces rapid depolymerization of a filament away from the nucleus. For each distribution of cell lengths, the model was run 500 times with the nucleus position recorded 30 minutes post infection.

Certain parameter means and standard deviations such as polymerization rate were obtained from previous measurements and publications [5]. Other parameters were estimated through an optimization scheme that minimized the root mean squared distance (RMSD) between the measured probability distribution of phage nuclei positioning in untreated, infected cells and the modeled. Initially, parameters were randomly mutated and RMSD was calculated. Using these initial points, the partial derivatives of RMSD with respect to each parameter are estimated and the next randomizations of each parameter are calculated until RMSD is minimized.

Surface plots were generated by linear interpolation between 200 points for any two parameters in order to visualize the RMSD parameter space (S2 Fig). The surface plot of polymerization rate vs depolymerization rate illustrates several interesting points about the model. Because RMSD is a measure of nuclei positions at the endpoints, it does not include any kinetic data from the model. This can be seen in the line of low RMSD (blue) which implies that any combination of polymerization rate and depolymerization rate that matches a certain net growth would produce similar results. This problem is avoided by having measured data for several parameters which helps constrain the polygon space.

## Supporting information

S1 Fig Microscopy of *P*. *aeruginosa* K2733 treated for 1 hr with various antibiotics without phage infection. Ciprofloxacin (CIP), daunorubicin (DAUN), rifampicin (RIF), gentamicin (GENT), ceftazidime (CEFT), mecillinam (MEC), meropenem (MERO), and piperacillin (PIP). Cell membrane strained with FM4-64 (red) and DNA stained with DAPI (blue). Scale bar represents 2 μm.
(TIF)

S2 Fig Surface plots of RMSD parameter spaces. RMSD between measured data and modeled results was used to optimize unknown parameters related to phuZ filament movement. Linear interpolation of 200 randomly sampled points for each parameter space allows for visualization of the relation between parameters. High RMSD (Yellow) is indicative of high deviation from measured data while low RMSD (Blue) indicates a good fit. Red dots indicate the values of parameters used in the final modeling.
(TIF)

S1 Dataset Phage nucleus counts and distance measurements. Data set is split by figure.
(XLSX)

## References

[1] Thomas J.A., Rolando M.R., Carroll C.A., Shen P.S., Belnap D.M., Weintraub S.T., et al., Characterization of Pseudomonas chlororaphis myovirus 201varphi2-1 via genomic sequencing, mass spectrometry, and electron microscopy. Virology, 2008. 376(2): p. 330–8. doi: 10.1016/j.virol.2008.04.004 18474389 PMC2577825

[2] Monson R., Foulds I., Foweraker J., Welch M., and Salmond G.P.C., The Pseudomonas aeruginosa generalized transducing phage phiPA3 is a new member of the phiKZ-like group of ’jumbo’ phages, and infects model laboratory strains and clinical isolates from cystic fibrosis patients. Microbiology (Reading), 2011. 157(Pt 3): p. 859–867. doi: 10.1099/mic.0.044701-0 21163841

[3] Krylov V.N., Dela Cruz D.M., Hertveldt K., and Ackermann H.W., “φKZ-like viruses”, a proposed new genus of myovirus bacteriophages. Archives of Virology, 2007. 152(10): p. 1955–1959. 17680323 10.1007/s00705-007-1037-7

[4] Mesyanzhinov V.V., Robben J., Grymonprez B., Kostyuchenko V.A., Bourkaltseva M.V., Sykilinda N.N., et al., The genome of bacteriophage phiKZ of Pseudomonas aeruginosa. J Mol Biol, 2002. 317(1): p. 1–19. doi: 10.1006/jmbi.2001.5396 11916376

[5] Chaikeeratisak V., Khanna K., Nguyen K.T., Sugie J., Egan M.E., Erb M.L., et al., Viral Capsid Trafficking along Treadmilling Tubulin Filaments in Bacteria. Cell, 2019. 177(7): p. 1771–1780 e12. doi: 10.1016/j.cell.2019.05.032 31199917 PMC7301877

[6] Chaikeeratisak V., Nguyen K., Egan M.E., Erb M.L., Vavilina A., and Pogliano J., The Phage Nucleus and Tubulin Spindle Are Conserved among Large Pseudomonas Phages. Cell Rep, 2017. 20(7): p. 1563–1571. doi: 10.1016/j.celrep.2017.07.064 28813669 PMC6028189

[7] Chaikeeratisak V., Nguyen K., Khanna K., Brilot A.F., Erb M.L., Coker J.K., et al., Assembly of a nucleus-like structure during viral replication in bacteria. Science, 2017. 355(6321): p. 194–197. doi: 10.1126/science.aal2130 28082593 PMC6028185

[8] Erb M.L., Kraemer J.A., Coker J.K., Chaikeeratisak V., Nonejuie P., Agard D.A., et al A bacteriophage tubulin harnesses dynamic instability to center DNA in infected cells. Elife, 2014. 3. 10.7554/eLife.03197 PMC4244570 25429514

[9] Erb M.L. and Pogliano J., Cytoskeletal proteins participate in conserved viral strategies across kingdoms of life. Curr Opin Microbiol, 2013. 16(6): p. 786–9. doi: 10.1016/j.mib.2013.08.007 24055040

[10] Chaikeeratisak V., Birkholz E.A., and Pogliano J., The Phage Nucleus and PhuZ Spindle: Defining Features of the Subcellular Organization and Speciation of Nucleus-Forming Jumbo Phages. Front Microbiol, 2021. 12: p. 641317. doi: 10.3389/fmicb.2021.641317 34326818 PMC8314001

[11] Laughlin T.G., Deep A., Prichard A.M., Seitz C., Gu Y., Enustun E., et al., Architecture and self-assembly of the jumbo bacteriophage nuclear shell. Nature, 2022. 608(7922): p. 429–435. doi: 10.1038/s41586-022-05013-4 35922510 PMC9365700

[12] Mendoza S.D., Nieweglowska E.S., Govindarajan S., Leon L.M., Berry J.D., Tiwari A., et al., A bacteriophage nucleus-like compartment shields DNA from CRISPR nucleases. Nature, 2020. 577(7789): p. 244–248. doi: 10.1038/s41586-019-1786-y 31819262 PMC6949375

[13] Malone L.M., Warring S.L., Jackson S.A., Warnecke C., Gardner P.P., Gumy L.F., et a;l., A jumbo phage that forms a nucleus-like structure evades CRISPR–Cas DNA targeting but is vulnerable to type III RNA-based immunity. Nature Microbiology, 2020. 5(1): p. 48–55. doi: 10.1038/s41564-019-0612-5 31819217

[14] Kraemer J.A., Erb M.L., Waddling C.A., Montabana E.A., Zehr E.A., Wang H., et al., A phage tubulin assembles dynamic filaments by an atypical mechanism to center viral DNA within the host cell. Cell, 2012. 149(7): p. 1488–99. doi: 10.1016/j.cell.2012.04.034 22726436 PMC3401054

[15] Adler H.I., Fisher W.D., Cohen A., and Hardigree A.A., MINIATURE escherichia coli CELLS DEFICIENT IN DNA. Proc Natl Acad Sci U S A, 1967. 57(2): p. 321–6. doi: 10.1073/pnas.57.2.321 16591472 PMC335508

[16] Rowlett V.W. and Margolin W., The bacterial Min system. Current Biology, 2013. 23(13): p. R553–R556. doi: 10.1016/j.cub.2013.05.024 23845239

[17] Merino-Salomón A., Babl L., and Schwille P., Self-organized protein patterns: The MinCDE and ParABS systems. Current Opinion in Cell Biology, 2021. 72: p. 106–115. doi: 10.1016/j.ceb.2021.07.001 34399108

[18] Rowlett V.W. and Margolin W., The Min system and other nucleoid-independent regulators of Z ring positioning. Frontiers in Microbiology, 2015. 6. doi: 10.3389/fmicb.2015.00478 26029202 PMC4429545

[19] Park K.-T., Villar M.T., Artigues A., and Lutkenhaus J., MinE conformational dynamics regulate membrane binding, MinD interaction, and Min oscillation. Proceedings of the National Academy of Sciences of the United States of America, 2017. 114(29): p. 7497–7504. doi: 10.1073/pnas.1707385114 28652337 PMC5530704

[20] de Boer P.A., Crossley R.E., Hand A.R., and Rothfield L.I., The MinD protein is a membrane ATPase required for the correct placement of the Escherichia coli division site. The EMBO journal, 1991. 10(13): p. 4371–4380. doi: 10.1002/j.1460-2075.1991.tb05015.x 1836760 PMC453190

[21] de Boer P.A., Advances in understanding E. coli cell fission. Curr Opin Microbiol, 2010. 13(6): p. 730–7. 20943430 10.1016/j.mib.2010.09.015 PMC2994968

[22] Margolin W., Bacterial Division: Journey to the Center of the Cell. Curr Biol, 2020. 30(3): p. R114–R116. doi: 10.1016/j.cub.2019.12.048 32017878

[23] CDC, Antibiotic Resistance Threats in the United States, C. U.S. Department of Health and Human Services, Editor. 2019.

[24] Chaudhry W.N., Concepcion-Acevedo J., Park T., Andleeb S., Bull J.J., and Levin B.R., Synergy and Order Effects of Antibiotics and Phages in Killing Pseudomonas aeruginosa Biofilms. PLoS One, 2017. 12(1): p. e0168615. doi: 10.1371/journal.pone.0168615 28076361 PMC5226664

[25] Taylor P.K., Yeung A.T.Y., and Hancock R.E.W., Antibiotic resistance in Pseudomonas aeruginosa biofilms: Towards the development of novel anti-biofilm therapies. Journal of Biotechnology, 2014. 191: p. 121–130. doi: 10.1016/j.jbiotec.2014.09.003 25240440

[26] Waters E.M., Neill D.R., Kaman B., Sahota J.S., Clokie M.R.J., Winstanley C., et al., Phage therapy is highly effective against chronic lung infections with Pseudomonas aeruginosa. Thorax, 2017. 72(7): p. 666–667. doi: 10.1136/thoraxjnl-2016-209265 28265031 PMC5520275

[27] Kortright K.E., Chan B.K., Koff J.L., and Turner P.E., Phage Therapy: A Renewed Approach to Combat Antibiotic-Resistant Bacteria. Cell Host & Microbe, 2019. 25(2): p. 219–232. doi: 10.1016/j.chom.2019.01.014 30763536

[28] Schooley R.T., Biswas B., Gill J.J., Hernandez-Morales A., Lancaster J., Lessor L., et al., Development and Use of Personalized Bacteriophage-Based Therapeutic Cocktails To Treat a Patient with a Disseminated Resistant Acinetobacter baumannii Infection. Antimicrob Agents Chemother, 2017. 61(10). 10.1128/AAC.00954-17 PMC5610518 28807909

[29] LaVergne S., Hamilton T., Biswas B., Kumaraswamy M., Schooley R.T., and Wooten D., Phage Therapy for a Multidrug-Resistant Acinetobacter baumannii Craniectomy Site Infection. Open Forum Infect Dis, 2018. 5(4): p. ofy064. doi: 10.1093/ofid/ofy064 29687015 PMC5905571

[30] Torres-Barcelo C., Arias-Sanchez F.I., Vasse M., Ramsayer J., Kaltz O., and Hochberg M.E., A window of opportunity to control the bacterial pathogen Pseudomonas aeruginosa combining antibiotics and phages. PLoS One, 2014. 9(9): p. e106628. doi: 10.1371/journal.pone.0106628 25259735 PMC4178015

[31] Gordillo Altamirano F.L. and Barr J.J., Phage Therapy in the Postantibiotic Era. Clin Microbiol Rev, 2019. 32(2). doi: 10.1128/CMR.00066-18 30651225 PMC6431132

[32] Oechslin F., Piccardi P., Mancini S., Gabard J., Moreillon P., Entenza J.M., et al., Synergistic Interaction Between Phage Therapy and Antibiotics Clears Pseudomonas Aeruginosa Infection in Endocarditis and Reduces Virulence. The Journal of infectious diseases, 2017. 215(5): p. 703–712. doi: 10.1093/infdis/jiw632 28007922 PMC5388299

[33] Burgess D.S., Use of pharmacokinetics and pharmacodynamics to optimize antimicrobial treatment of Pseudomonas aeruginosa infections. Clin Infect Dis, 2005. 40 **Suppl** 2: p. S99–104. doi: 10.1086/426189 15712103

[34] Uchiyama J., Shigehisa R., Nasukawa T., Mizukami K., Takemura-Uchiyama I., Ujihara T., et al., Piperacillin and ceftazidime produce the strongest synergistic phage–antibiotic effect in Pseudomonas aeruginosa. Archives of Virology, 2018. 163(7): p. 1941–1948. doi: 10.1007/s00705-018-3811-0 29550930

[35] Nonejuie P., Burkart M., Pogliano K., and Pogliano J., Bacterial cytological profiling rapidly identifies the cellular pathways targeted by antibacterial molecules. Proc Natl Acad Sci U S A, 2013. 110(40): p. 16169–74. doi: 10.1073/pnas.1311066110 24046367 PMC3791758

[36] Lamsa A., Liu W.T., Dorrestein P.C., and Pogliano K., The Bacillus subtilis cannibalism toxin SDP collapses the proton motive force and induces autolysis. Mol Microbiol, 2012. 84(3): p. 486–500. doi: 10.1111/j.1365-2958.2012.08038.x 22469514 PMC3839633

[37] Lamsa A., Lopez-Garrido J., Quach D., Riley E.P., Pogliano J., and Pogliano K., Rapid Inhibition Profiling in Bacillus subtilis to Identify the Mechanism of Action of New Antimicrobials. ACS chemical biology, 2016. 11(8): p. 2222–2231. doi: 10.1021/acschembio.5b01050 27193499 PMC5459310

[38] Quach D.T., Sakoulas G., Nizet V., Pogliano J., and Pogliano K., Bacterial Cytological Profiling (BCP) as a Rapid and Accurate Antimicrobial Susceptibility Testing Method for Staphylococcus aureus. EBioMedicine, 2016. 4: p. 95–103. doi: 10.1016/j.ebiom.2016.01.020 26981574 PMC4776060

[39] Htoo H.H., Brumage L., Chaikeeratisak V., Tsunemoto H., Sugie J., Tribuddharat C., et al., Bacterial Cytological Profiling as a Tool To Study Mechanisms of Action of Antibiotics That Are Active against Acinetobacter baumannii. Antimicrob Agents Chemother, 2019. 63(4). doi: 10.1128/AAC.02310-18 30745382 PMC6437480

[40] Ceyssens P.-J., Minakhin L., Van den Bossche A., Yakunina M., Klimuk E., Blasdel B., et al., Development of giant bacteriophage ϕKZ is independent of the host transcription apparatus. Journal of virology, 2014. 88(18): p. 10501–10510. 24965474 10.1128/JVI.01347-14 PMC4178840

[41] deYmartín Garrido N., Orekhova M., Lai Wan Loong Y.T.E., Litvinova A., Ramlaul K., Artamonova T., et al., Structure of the bacteriophage PhiKZ non-virion RNA polymerase. Nucleic acids research, 2021. 49(13): p. 7732–7739. doi: 10.1093/nar/gkab539 34181731 PMC8287921

[42] Kohanski M.A., Dwyer D.J., and Collins J.J., How antibiotics kill bacteria: from targets to networks. Nat Rev Microbiol, 2010. 8(6): p. 423–35. doi: 10.1038/nrmicro2333 20440275 PMC2896384

[43] Lai G.C., Cho H., and Bernhardt T.G., The mecillinam resistome reveals a role for peptidoglycan endopeptidases in stimulating cell wall synthesis in Escherichia coli. PLoS Genet, 2017. 13(7): p. e1006934. doi: 10.1371/journal.pgen.1006934 28749938 PMC5549755

[44] Chen Y., Milam S.L., and Erickson H.P., SulA inhibits assembly of FtsZ by a simple sequestration mechanism. Biochemistry, 2012. 51(14): p. 3100–3109. doi: 10.1021/bi201669d 22432817 PMC3518438

[45] Drew K.R. and Pogliano J., Dynamic instability-driven centering/segregating mechanism in bacteria. Proc Natl Acad Sci U S A, 2011. 108(27): p. 11075–80. doi: 10.1073/pnas.1018724108 21685333 PMC3131362

[46] Gerdes K., Howard M., and Szardenings F., Pushing and pulling in prokaryotic DNA segregation. Cell, 2010. 141(6): p. 927–42. doi: 10.1016/j.cell.2010.05.033 20550930

[47] Lasker K., Mann T.H., and Shapiro L., An intracellular compass spatially coordinates cell cycle modules in Caulobacter crescentus. Curr Opin Microbiol, 2016. 33: p. 131–139. doi: 10.1016/j.mib.2016.06.007 27517351 PMC5069156

[48] Surovtsev I.V. and Jacobs-Wagner C., Subcellular Organization: A Critical Feature of Bacterial Cell Replication. Cell, 2018. 172(6): p. 1271–1293. doi: 10.1016/j.cell.2018.01.014 29522747 PMC5870143

[49] Sanchez A.D., Branon T.C., Cote L.E., Papagiannakis A., Liang X., Pickett M.A., et al., Proximity labeling reveals non-centrosomal microtubule-organizing center components required for microtubule growth and localization. Curr Biol, 2021. 31(16): p. 3586–3600 e11. doi: 10.1016/j.cub.2021.06.021 34242576 PMC8478408

[50] Birkholz E.A., Laughlin T.G., Suslov S., Armbruster E., Lee J., Wittmann J., et al., A Cytoskeletal Vortex Drives Phage Nucleus Rotation During Jumbo Phage Replication in &lt;em&gt;E. coli&lt;/em&gt. bioRxiv, 2021: p. 2021.10.25.465362. 10.1016/j.celrep.2022.111179 PMC9891218 35977483

[51] Guan J., Oromí-Bosch A., Mendoza S.D., Karambelkar S., Berry J., and Bondy-Denomy J., RNA targeting with CRISPR-Cas13a facilitates bacteriophage genome engineering. bioRxiv, 2022: p. 2022.02.14.480438. 10.1038/s41564-022-01243-4 PMC9722621 36316452

