# Supplementary figures and images for: Bacterial cytological profiling reveals interactions between jumbo phage φKZ infection and cell wall active antibiotics in Pseudomonas aeruginosa
Source: PLoS One. 2023 Jul 7;18(7):e0280070. doi: 10.1371/journal.pone.0280070 (PMC10328376; doi:10.1371/journal.pone.0280070)

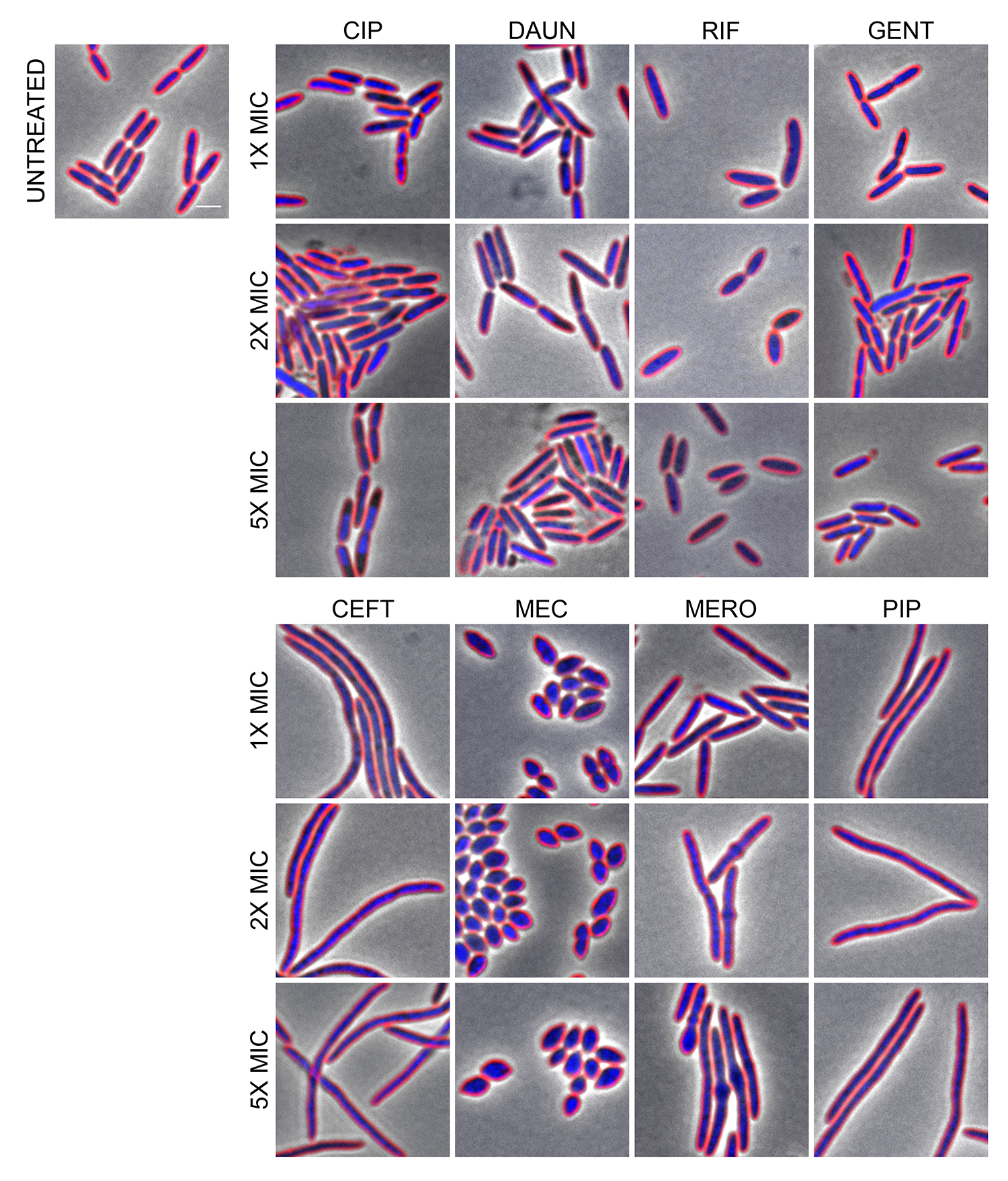

Supplement: S1 Fig — Ciprofloxacin (CIP), daunorubicin (DAUN), rifampicin (RIF), gentamicin (GENT), ceftazidime (CEFT), mecillinam (MEC), meropenem (MERO), and piperacillin (PIP). Cell membrane strained with FM4-64 (red) and DNA stained with DAPI (blue). Scale bar represents 2 μm. (TIF) [file pone.0280070.s001.tif]

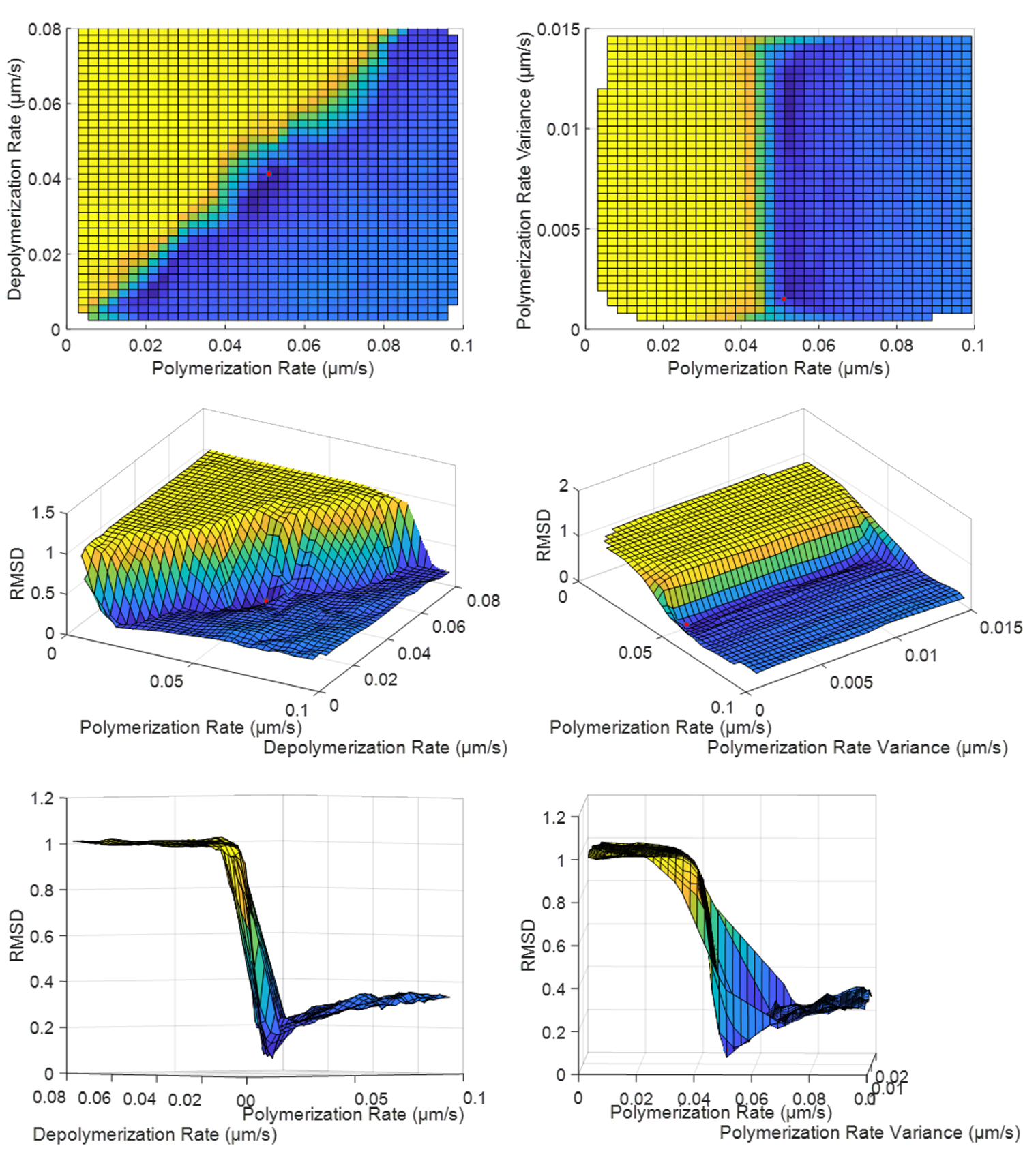

Supplement: S2 Fig — RMSD between measured data and modeled results was used to optimize unknown parameters related to phuZ filament movement. Linear interpolation of 200 randomly sampled points for each parameter space allows for visualization of the relation between parameters. High RMSD (Yellow) is indicative of high deviation from measured data while low RMSD (Blue) indicates a good fit. Red dots indicate the values of parameters used in the final modeling. (TIF) [file pone.0280070.s002.tif]
